# Diverse modes of reproduction in the marine free-living ciliate *Glauconema trihymene*

**DOI:** 10.1186/1471-2180-10-108

**Published:** 2010-04-13

**Authors:** Hongan Long, Rebecca A Zufall

**Affiliations:** 1 Department of Biology and Biochemistry, University of Houston, Houston TX, 77204-5001 USA

## Abstract

**Background:**

Most free-living ciliates reproduce by equal fission or budding during vegetative growth. In certain ciliates, reproduction occurs inside the cyst wall, *viz*. reproductive cysts, but more complex reproductive strategies have generally been thought to be confined to parasitic or symbiotic species, e.g. *Radiophrya *spp.

**Results:**

In addition to equal fission, asymmetric binary division and reproductive cysts were discovered in the free-living bacterivorous scuticociliate *Glauconema trihymene *Thompson, 1966. Asymmetric division is an innate physiological state that can be induced by sufficient food, and the higher the food concentration, the longer the asymmetric division persists. During asymmetric division, nuclear and somatic structures divide with transiently arrested cytokinesis and variable positioning of macronuclei. Phylogenetic analysis, based on the small subunit of ribosomal DNA (SSU rDNA) sequences, showed that the *G. trihymene *isolate studied here nests with typical scuticociliates and is paraphyletic to both the symbiotic apostome and astome ciliates, some of which also produce progeny by asymmetric division.

**Conclusions:**

The asymmetric division in *G. trihymene *has no precedent among undisturbed free-living ciliates. The coexistence of multiple modes of reproduction may represent a previously undescribed reproductive strategy for ciliates living on food patches in coastal waters. This may also be indicative of similar reproductive strategies among other polyphenic ciliates, which have not been intensively studied. Asymmetric division provides a special opportunity for studying ciliates' phenotypic plasticity and may also illuminate the origins of multicellularity.

## Background

Ciliates are a diverse group of unicellular eukaryotes characterized by two kinds of nuclei in each cell: a germline micronucleus and a somatic macronucleus. Free-living ciliates are known to exhibit diversity in modes of reproduction [1-3]. Most of these reproductive modes include equal fission or budding. In certain ciliates, including *Tetrahymena patula *and *Colpoda inflata*, reproduction can also occur inside the cyst wall, *viz*. reproductive cysts [3,4].

Symbiotic ciliates like the astome ciliates, e.g., *Radiophrya *spp., and certain apostome ciliates, e.g., *Polyspira *spp., reproduce by forming cell chains, also called catenoid colonies, which are usually brought about by repeated asymmetric division without separation of the resulting filial products [3,5]. Some *Tetrahymena*, such as temperature-sensitive cytokinesis-arrested mutants of *T. thermophila*- strain cdaC, and *T. pyriformis *also showed similar cell chains at high temperature [6,7] and similar morphotypes were also recently reported in the non-reproductive artificial lethal mutants of *T. thermophila *[8]. However, no free-living ciliates have been reported to form cell chains in response to food (bacteria) concentration.

During early and late phases of equal fission, most ciliates share certain features, such as common positioning of the macronucleus and the micronucleus, synchronization of macronuclear amitosis and fission furrow, and a specific and well defined dividing size [9-11]. It is generally assumed that if food density meets requirements of both cell development and division, the daughter cells will be identical, so after division, the two daughter cells could not be differentiated from each other [12-14].

However, ciliates from the same single cell isolate were reported to have high diversity in physiological states, such as cell size and volume, growth rate, feeding and digestion [15-18], and certain ciliates even develop highly unique physiological strategies to maximally adapt to their habitats. For example, after feeding on the cryptomonad *Geminigera cryophila*, the mixotrophic red-tide-causing ciliate *Myrionecta rubra *retains the prey organelles, which continue to function in the ciliate for up to 30 days [19,20]. Comprehensive analysis of physiological state changes of ciliates usually requires monitoring of individuals for a relatively long period and therefore is rarely conducted [15]. Most ciliates are currently unculturable or swim too fast for microscopic observation, further hindering such analyses.

In this study, we describe a series of reproductive strategies that have been previously unknown in free-living ciliates. These types of reproduction occurred in all newly established cultures of *G. trihymene*, a free-living scuticociliate belonging to the class Oligohymenophorea, which also includes *Tetrahymena *and *Paramecium*. The division processes and the relationship between persistence time of asymmetric divisions and bacteria concentrations are described, and an updated life cycle and phylogenetic position of *G. trihymene *are presented.

## Results

### Natural History of *G. trihymene*

**Figure 1:**
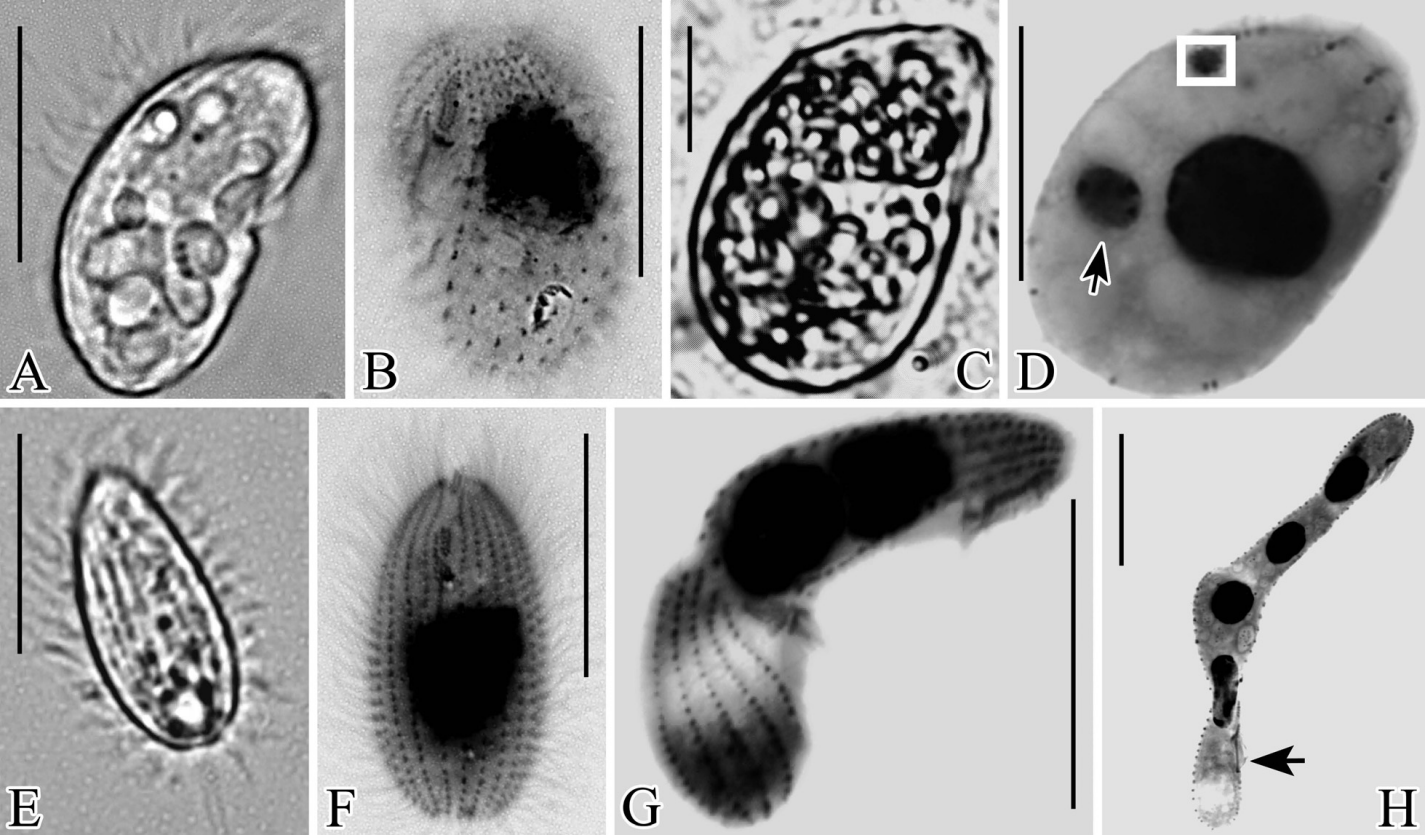
***G. trihymene *morphotypes**. A, C, E were from living cells; B, D, F- H were from protargol impregnated specimens. A, B. Lateral and ventral view of trophonts. C. A well-fed trophont. D. One probable asymmetric divider. Arrow marks the smaller macronucleus. The white square frame marks the micronucleus from a different plane of focus. The smaller macronucleus differs from the micronucleus by having many nucleoli. E, F. Ventral view of tomites. G. One asymmetric divider with two displaced macronuclei. H. One long asymmetric divider, probably releasing one trophont (arrow). Scale bars: A-H: 25 μm.

The *G. trihymene *isolate described here, collected in Hong Kong, is free-living and bacterivorous. It has a polyphenic life cycle that includes the following three previously described stages [21,22]: trophont, reniform, the feeding and division stage, mostly 35 × 20 μm *in vivo *(Figure 1A, B); tomite, the dispersion and fast-swimming stage in response to starvation, with a spindle-shaped cell, mostly 30 × 15 μm *in vivo *(Figure 1E, F); resting cyst, mostly rounded, dormant stage during trophic depletion, ca. 20 μm in diameter. Like other free-living ciliates, *G. trihymene *has a transcriptionally active macronucleus and a germline micronucleus. The infraciliature and buccal apparatus are the same as in previous reports, however, we found the life cycle was much more complicated and included two reproductive modes new to scuticociliates, asymmetric division and reproductive cysts.

### Processes of asymmetric division in young cultures

**Figure 2:**
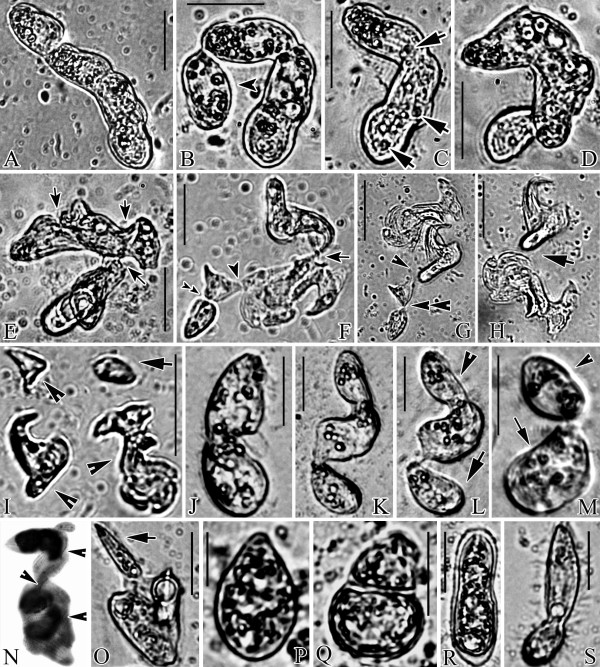
**Division processes of two *G. trihymene *asymmetric dividers in young cultures (A-I, J-M), other asymmetric dividers in young (N) and old cultures (O, S), and reproductive cysts (P-R)**. A. One four-subcell asymmetric divider. B. The first asymmetric division. Arrowhead marks the trophont to be released. C-E. The new asymmetric divider gradually became highly deformed and many cleavage furrows appeared (arrows in E). Note the three contractile vacuoles in C (arrows). F. The arrowhead, double-arrowheads and arrow show the sites of the second, third and fourth cleavage furrows respectively. G. The second asymmetric division is completed at the arrowhead. The double arrowheads show the furrow that will shortly be broken in the third asymmetric division. H. The trophont resulting from the completion of the third asymmetric division has swum out of the field of view. The fourth asymmetric division has just been completed near the arrow, at a site corresponding to the furrow indicated by the arrow in F. I. Three new asymmetric dividers (arrowheads) and one trophont (arrow) were present by the end of the fourth asymmetric division. J. One two-subcell asymmetric divider. K, L. After elongation, the first asymmetric division produced one trophont (arrow in L) and one asymmetric divider (arrowhead in L). M. The second asymmetric division, producing one trophont (arrowhead) and another asymmetric divider (arrow). N. Arrowheads mark oral apparatuses (after protargol). O. One asymmetric divider releasing a tomite (arrow). P, Q. The division process of reproductive cysts. R. Another asymmetric divider forming a cyst wall. S. An asymmetric divider resembling a dividing tomite. Scale bars: A-H: 50 μm; I: 100 μm; J-M, O-S: 25 μm.

Many slowly moving, well-fed trophonts (Figure 1C) appeared within 24 hours after inoculation with tomites in cultures of wheat grain medium. In all of the cultures, a trophont underwent a cell division, but cytokinesis was arrested prior to completion, creating a unit consisting of two cells, now called "subcells" because of their failure to separate. Typically, each of the two connected subcells later underwent a second transverse division, resulting in a chain of four subcells, each with a macronucleus, an oral apparatus, and a contractile vacuole (Figures 1H; 2A). We define these chains of subcells as asymmetric dividers. Asymmetric dividers vary in sizes from 30 × 15 μm to 180 × 30 μm *in vivo*, have diverse shapes consisting of chains of 2-4 subcells (Figures 1G, H; 2A, J, O) and give rise to two filial cells that could be morphologically differentiated from each other after each division. Similar asymmetric dividers were also repeatedly found in different cultures, though the sizes varied with media type. Up to 4 macronuclei were found in the cytoplasm of each asymmetric divider (Figure 1H). Most undisturbed asymmetric dividers attached to the bottom of Petri dishes, moved very slowly or stayed immobile and had two or more rounded contractile vacuoles, pulsating with different frequencies (arrows in Figure 2C). The number of asymmetric dividers in the cultures increased with time from appearance of the first asymmetric divider.

Several asymmetric dividers were continuously followed on inverted microscopes. Two typical division processes of asymmetric dividers in young cultures (the 3^rd ^or 4^th ^day after inoculation) are described in detail (Figure 2A-M):

The first division of one long asymmetric divider (Figure 2A) occurred about two hours after it was found. During this first division, the cell's most anterior part was released (the anterior and posterior ends were judged from the moving direction and posterior position of the contractile vacuoles) as a trophont and quickly swam away (Figure 2B, arrowhead). The larger posterior part became a new asymmetric divider (Figure 2C), which then deformed so much that no clear body axis could be determined (Figure 2D, E). The division types (transverse or longitudinal) were thus not easily categorized and many cleavage furrows appeared (Figure 2E, arrows). The second asymmetric division occurred through disjuncture or fission at the most mature cleavage furrow (Figure 2F, G, arrowheads). Then after about three minutes, the other two furrows broke (Figure 2F-H, double-arrowheads, arrows). Finally, three new asymmetric dividers, which were also slowly moving or immobile and continued dividing highly unequally (Figure 2I, arrowheads), and one trophont (Figure 2I, arrow) were produced. The entire process described above occurred over the course of 22 hours.

The most common asymmetric dividers in young cultures had two subcells (Figure 2J), which divided over the course of 6 hours. The division process (Figure 2K-M) was similar to the one described above in that the first division yielded one active trophont (Figure 2L, arrow) and one new asymmetric divider (Figure 2L, arrowhead). After that, however, the newly formed asymmetric divider divided into one trophont (Figure 2M, arrowhead) and one new asymmetric divider (Figure 2M, arrow), which became deformed and continued dividing highly unequally. During each division, the asymmetric dividers either produced one trophont and one new asymmetric divider (as shown in Figure 2B, L, M) or two new asymmetric dividers (Figure 2G, H).

### Asymmetric dividers and reproductive cysts in old cultures

When bacteria were depleted, most trophonts transformed into tomites and the cultures were termed "old". In the soil extract medium with various bacteria concentrations, this usually occurred between 141 and 175 hours after inoculation (Table 1). In old cultures, asymmetric division continued, but produced tomites instead of trophonts (Figure 2O, arrow). Small asymmetric dividers producing tomites sometimes looked like dividing tomites (Figure 2S). Some asymmetric dividers were also found to die and were observed with a large central vacuole. Reproductive cysts were also found: some asymmetric dividers developed transparent cyst walls and continued to divide unequally one or two times inside the cyst walls (Figure 2P-R).

**Table 1:** Average first appearance time of tomites in three different concentrations of bacteria in the soil extract medium (four replicates for each concentration).

Bacterial concentrations of cultures	Tomite first appearance time (hours after inoculation)
0.01×	141.5
0.1×	168.1
1×	174.9

### Somatic and nuclear characteristics of asymmetric dividers after protargol impregnation

Some asymmetric dividers had similar body shape to trophonts, except having two highly unequal macronuclei (Figure 1D). Macronuclear divisions could also happen several times before the completion of cytokinesis, producing up to 4 macronuclei in the same cytoplasm (Figure 1H). The positioning of macronuclei was highly variable even if the cleavage furrows were clearly formed (Figures 1G, H; 2N). Usually more than two buccal apparatuses were present in bigger asymmetric dividers (Figure 2N, arrowheads).

### Is asymmetric division a cultural artifact?

Actively dividing asymmetric dividers were found in all wheat grain medium cultures and cultures with bacterial suspensions in the soil extract medium, as well as cultures started with single cells as inocula. Even though the seawater for cultures was changed twice (natural seawater from coastal areas of Galveston TX, USA), asymmetric dividers were found in all cultures under study. Asymmetric dividers also showed up in early cultures of another seven *G. trihymene *isolates collected from coastal areas of Texas, USA (Table 2). The regularity with which asymmetric dividers appear and their consistent response to bacterial concentrations (see below) suggest that these asymmetric dividers are not cultural artifacts.

**Table 2:** *Glauconema trihymene *isolates with asymmetric divisions.

Strain Name	Collecting Site	Collection Date	Habitat
PRA-270	Hong Kong	08/20/2007	Rinsing/crab
PB508151	Port Bolivar, TX	08/15/2009	Sea lettuce
PB508152	Port Bolivar, TX	08/15/2009	Sea lettuce
PB508293	Port Bolivar, TX	08/29/2009	Sea lettuce
PI108293	Pelican Island, TX	08/29/2009	Sea lettuce
PI108294	Pelican Island, TX	08/29/2009	Sea lettuce
PI608291	Pelican Island, TX	08/29/2009	Sea lettuce
QP76	Quintana Park, Freeport, TX	10/24/2009	Sea lettuce

### Relationship between asymmetric dividers and food abundance

**Figure 3:**
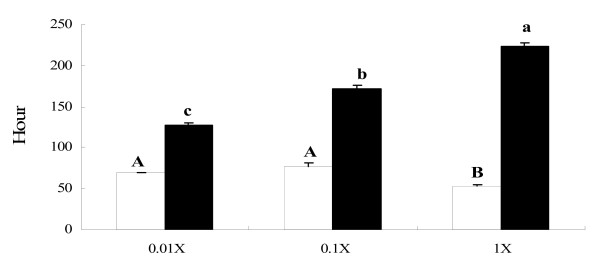
**First appearance time and duration of persistence of asymmetric divisions**. The time of appearance of the first asymmetric divider in the newly inoculated cultures (hollow bars) and the duration of persistence of asymmetric divisions after the appearance of the first asymmetric divider (filled bars) were noted for cells maintained in the Erd-Schreiber soil extract cultures with one of three different bacterial concentrations. Appearance time of first asymmetric dividers and persistence time of asymmetric divisions were analyzed independently. Error bars: standard error. Levels not connected by the same letter are significantly different (P < 0.05).

All asymmetric dividers first appeared on the 3^rd ^to 4^th ^day (51-93 hours) (Figure 3, hollow bars) after inoculation of tomites into three bacterial concentrations. The earliest asymmetric dividers appeared in the cultures with the highest bacterial concentration (P < 0.05, Oneway ANOVA; Figure 3, hollow bar B), on average 54 hours after inoculation. There was no significant difference between the time of first appearance of asymmetric dividers in the other cultures (P > 0.05, Oneway ANOVA; Figure 3, hollow bars A).

After the first asymmetric dividers appeared in each culture, they were checked every 12 hours until no asymmetric dividers remained. The time interval between first appearance of asymmetric dividers and the time when no asymmetric divider could be found was recorded for each culture (Figure 3, filled bars). The time during which no asymmetric divider could be found was probably the stationary phase, when cells had run out of food so that they could not divide at all. This time interval, reflecting the total time of asymmetric divisions in each culture, was found to increase with bacterial concentration (Figure 3, filled bars, a-c; Oneway ANOVA, P < 0.05).

### Phylogenetic position of *Glauconema trihymene*

**Figure 4:**
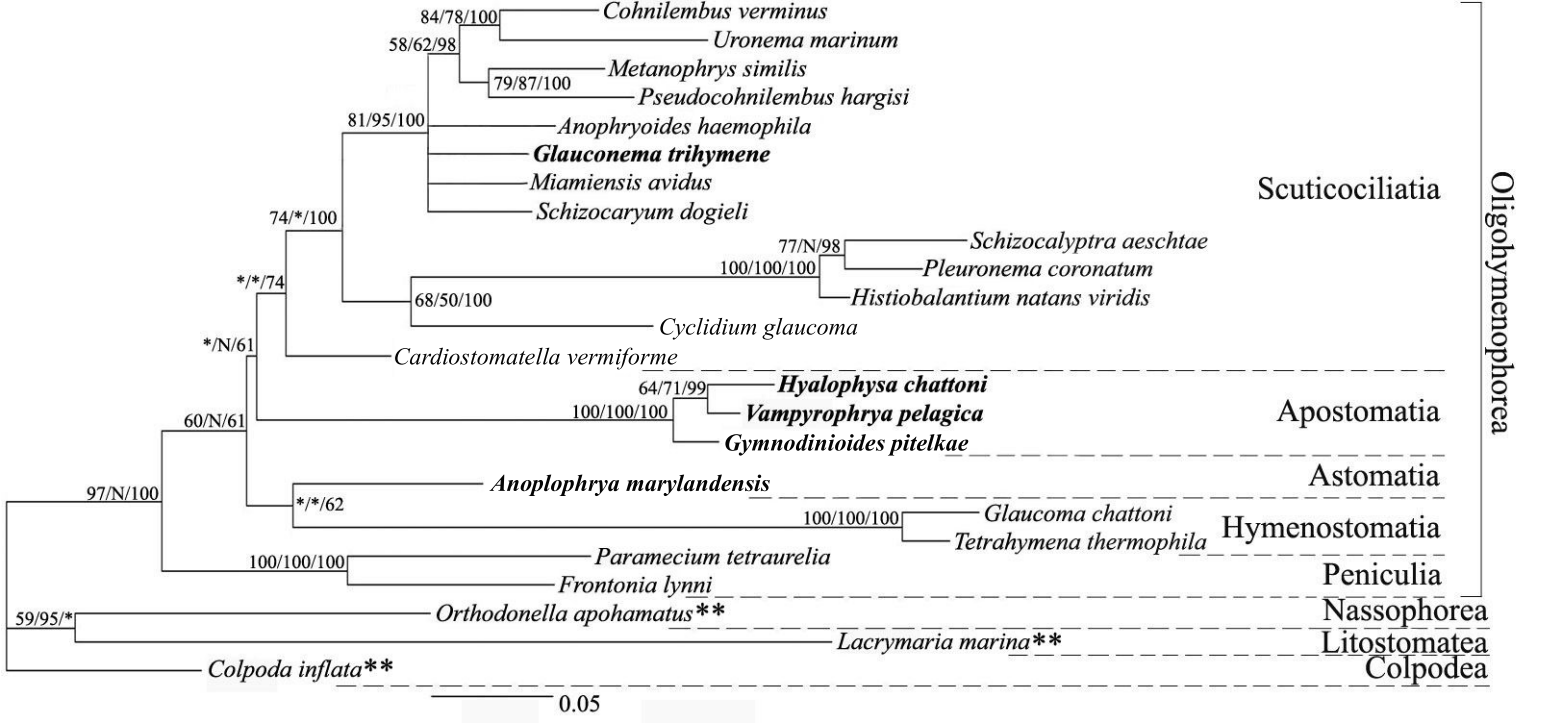
**Phylogenetic position of *G. trihymene***. Maximum likelihood tree topology and branch lengths, rooted with species marked with **. Support for clades is indicated by ML boostrap/MP bootstrap/MB posterior probabilities. N indicates that this clade was not found in the given analysis and asterisks indicate clades with support of less than 50%. Nodes with <50% support in all methods are shown as a polytomy. Scale bar: 5 substitutions per 100 nucleotide positions.

Maximum likelihood, maximum parsimony and Baysian trees, inferred from 18S SSU rDNA sequences, all show that *G. trihymene *(Hong Kong isolate) groups with typical scuticociliates, like *Anophryoides haemophila *and *Miamiensis avidus *(Figure 4). The Hong Kong isolate shares 81.2% DNA pair-wise identity with a previously submitted *G. trihymene *sequence [GenBank Accession No.: AY169274].

## Discussion

### Updated life cycle of *G. trihymene *during vegetative growth

**Figure 5:**
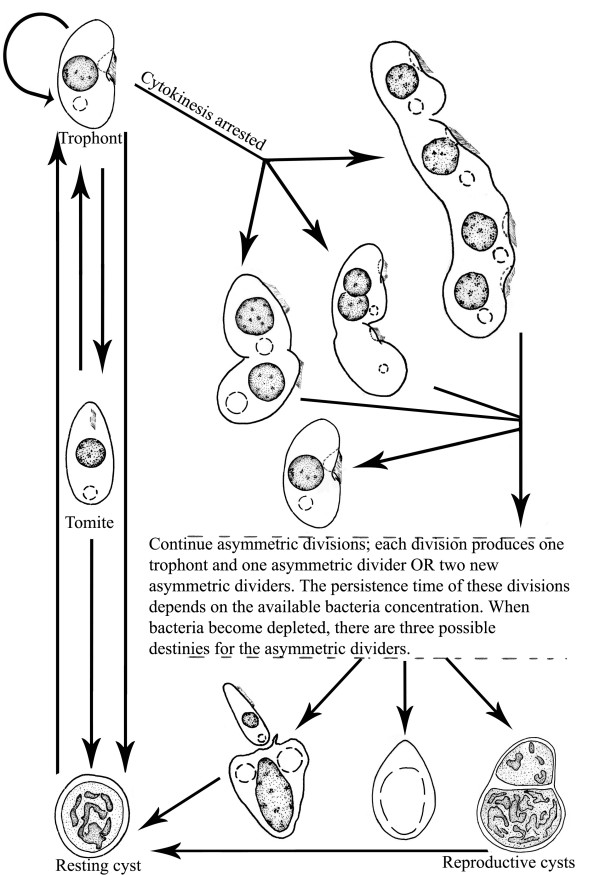
**Updated life cycle of *G. trihymene *in vegetative growth**. This is generalized from continuous microscopy and observation of specimens after protargol impregnation. Note the first asymmetric dividers (probably more than three morphotypes) with different sizes and shapes in early cultures developed through the arrest of cytokinesis in some trophonts. Drawings are not strictly to scale. Information on micronuclei is not available.

The life cycle during vegetative growth of *G. trihymene *is generalized in Figure 5, based on previous and current studies [21,22]. The life cycle has multiple stages, as is typical in polyphenic ciliates. These life stages could be highly diverse and complex, depending on the total number of asymmetric divider morphotypes and food concentration. For simplification and clarity, most intermediate asymmetric dividers are not shown in Figure 5.

Some free-living ciliates, for example, *Tetrahymena pyriformis*, produce maximal progeny cells by shifting their physiological states during starvation [23]. Similarly, *G. trihymene *produces progeny cells by combining three reproductive modes: asymmetric division, reproductive cysts and equal fission. In addition, this is the first report of reproductive cysts in scuticociliates, though they are not uncommonly found in certain ciliate genera, like *Colpoda *and *Tetrahymena *[4]. If each morphotype of asymmetric dividers could be deemed as one life stage, which could probably be the case as many similar or continuous asymmetric divider morphotypes were repeatedly found in cultures with different "age" or media, then the updated life cycle of *G. trihymene *might rival most known life cycles of free-living ciliates in complexity (Figure 5). *G. trihymene *thus provides a special opportunity for studying ciliate polyphenism.

Although *G. trihymene *was first discovered early in 1966, it was believed to reproduce only by equal fission during vegetative growth [21,22]. One reason for the persistence of this narrow view of *G. trihymene *reproduction is that, to date, few studies have been conducted on *G. trihymene *and they have mainly focused on morphology or systematics rather than reproduction dynamics [21,22]. Secondly, some of the reproductive forms appear only under particular food conditions, for example, in the Hong Kong isolate, asymmetric dividers appeared on the 3^rd ^or 4^th ^day after inoculation, when bacterial supply was high and disappeared soon after the appearance of tomites. The disappearance of asymmetric dividers was probably associated with the transition from exponential culture growth to the stationary phase. Third, the relative immobility and irregular body shapes of most asymmetric dividers (Figures 1G, H; 2E, N), could cause them to be mistaken as cultural artifacts or debris. Lastly, some asymmetric dividers are easily mistaken as conjugating cells or equal binary dividers, if observed on low magnifications (<100×) (Figure 2J). Thus, it is no wonder that these usually large, irregularly shaped asymmetric dividers were unreported until this study.

The class Oligohymenophorea, to which all scuticociliates and the well-known *Tetrahymena *and *Paramecium *belong, contains highly diverse species [24], but only a few model species, such as *Tetrahymena thermophila *and *Paramecium tetraurelia*, are under intensive biological study. Most members of Oligohymenophorea, especially the marine species, are limited to taxonomic and systematic studies or are undescribed [2,25]. We predict that as life histories of more species are closely examined, much more diversity in reproductive strategies will be discovered among free-living protists.

### Proposed ecological roles of various life cycle stages

The high feeding efficiency, slow movement and arrested cytokinesis observed in *G. trihymene *asymmetric dividers may be advantageous. Based on the results of our culturing experiments, we conclude that asymmetric dividers are innate physiological states of *G. trihymene*, which can be induced to occur in bacteria-sufficient media. Cells with asymmetric divisions may ingest more food than those without; most asymmetric dividers had many oral apparatuses with oral membranes beating quickly. They may be able to consume as many bacteria as several trophonts in the same period of time (Figure 2N, arrowheads). In addition, the relative immobility of these asymmetric dividers may minimize their energy consumption [26]. The arrested cytokinesis could also save energy for asymmetric dividers, compared with equal dividers.

We propose the following ecological scenario that comes about as *G. trihymene *with a capacity for asymmetric divisions explores its surrounding environment. Suppose one *G. trihymene *trophont finds a food patch with plenty of bacteria, but also with many other bacteria-feeding protists. To avoid being a loser in this resource exploitation competition, for 2-3 days *G. trihymene *vigorously feeds on bacteria and divides equally. While plenty of bacteria remain, some trophonts asymmetrically divide, producing trophonts and more asymmetric dividers. When the food patch is nearly exhausted, most trophonts transform into tomites, and the asymmetric dividers instead of producing trophonts, produce tomites. After most of the bacteria are consumed, most tomites become resting cysts. Asymmetric dividers secrete a cyst wall and continue dividing inside, producing reproductive cysts, which ultimately become resting cysts. Some tomites transformed from trophonts or released by asymmetric dividers swim rapidly to seek more food patches, transforming back into trophonts when they find new food patches and repeating the above processes. The quickly dispersing tomites, the tolerating resting cysts, and the diverse reproductive strategy may enable *G. trihymene *to identify and dominate enough food patches and survive in the coastal water community.

### Phylogenetic position of *G. trihymene*, and asymmetric division

*G. trihymene *groups with typical scuticociliates with high bootstrap support and posterior probability, though the precise relationships within the clades remain unresolved (Figure 4). In addition, *G. trihymene *has high SSU rDNA pair-wise identity with *Anophryoides haemophila *(96%), the scuticociliate causing the "Bumper car disease" of American lobsters and *Miamiensis avidus *(96%), a polyphenic, parasitic ciliate, which causes diseases in fish [27,28]. Our result supports the monophyly of scuticociliatia, despite what was found in earlier studies utilizing a previously reported *G. trihymene *SSU rDNA sequence [GenBank Accession No.: AY169274] [29,30], which we believe to be erroneous. AY169274 shares great similarity with SSU sequences of some flagellates, e.g. it has 96% identity with the 18S rDNA sequences of the nanoflagellate *Spumella *sp. GOT220 [GenBank Accession No.: EF027354]. In line with our interpretation, the most recent study on morphology and morphogenesis of *G. trihymene *(performed by the same group that submitted the previous Gt SSU rDNA sequence) showed that it is indeed a typical scuticociliate [22].

Asymmetric divisions, similar to those in *G. trihymene*, occur in certain apostome and many astome ciliates (see phylogenetic position in Figure 4), though the details of division had never been studied using continuous microscopy [5]. Such asymmetric dividers were called catenoid colonies in these host-dependent ciliates. Asymmetric dividers were so named in the present study to emphasize the difference between the two filial cells. As in the asymmetric division of *G. trihymene *in Figure 2A, long cell chains in the parasitic and commensal astome and apsotome ciliates are formed by repeated incomplete divisions without separation of the resulting filial products, after which some subcells are fully or partially pinched off. These subcells require subsequent metamorphosis to regain the form typical of the normal trophont stage of the life cycle [3,5].

The results of the phylogenetic analysis suggest that complex life cycles including asymmetric division are either 1) an ancestral feature of these three groups that has been modified, lost, or not yet discovered in other free-living species, or 2) a convergent trait that has arisen multiple times independently in these closely related taxa.

### Asymmetric division: one clue to multicellularity?

The colonial flagellate hypothesis, claiming that flagellated protists living as colonies evolved into the first animals, has inspired extensive productive exploration on the origin of multicellularity [31-34]. The asymmetric division of *G. trihymene *serves as an alternative mechanism through which ciliates may have led to a multicellular form: a multicellular form could arise by a ciliate with one macronucleus and one micronucleus subdividing itself as a result of growth followed by arrested cytokinesis. It should be noted, however, that such asymmetric division does not result in different developmental fates akin to truly multicellular ciliate species, such as *Zoothamnium alternans *[35,36].

As is shown in this study, asymmetric dividers produce new asymmetric dividers and trophonts by successive asymmetric divisions, in favorable conditions, and the more available food, the longer the asymmetric divisions persisted (Figure 3, filled bars). If asymmetric dividers lived in consistently bacteria-rich environments for a long time, they might retain the multicellular form, but lose the ability to produce trophonts or tomites. Bacteria-rich environments were common in the ancient ocean, which had very different chemistry from that of today's [37,38]. Thus, it is possible that some multicellular organisms, which have not yet been discovered or have since gone extinct, originated from certain asymmetric dividers of ciliates.

## Conclusions

Diverse reproductive modes in *G. trihymene *were unexpectedly discovered. This study is the first to report asymmetric division and reproductive cysts in scuticociliates. In addition, the presence of multiple reproductive modes is a previously undescribed reproductive strategy for ciliates living on food patches in coastal waters. The asymmetric dividers may give insight into possible origins of multicellularity and provide a special opportunity for studying ciliate polyphenism. We predict that asymmetric division and other reproductive strategies will be discovered in other polyphenic protists through more intensive study.

## Methods

### Sampling and identifying *G. trihymene*

*G. trihymene *PRA-270 was isolated with a fine pipette from a seawater rinse of a newly dead crab (species unknown) collected from a sand beach near the pier of Hong Kong University of Science and Technology, Clear Water Bay, Hong Kong (22°20' N; 114°17' E) on August 20, 2007. The salinity was about 33‰, temperature 26°C, and pH 8.1. The cultures used in this study were derived from a single *G. trihymene *cell of the Hong Kong isolate. Seven other isolates were collected from Texas coastal areas (Table 2). The salinity was about 33‰ and temperature ranged from 23 to 31°C. Trophonts and tomites of *G. trihymene *were observed *in vivo *first using a stereomicroscope and then an epi-fluorescence microscope at 100-1000×. The nuclear apparatuses and infraciliature were revealed by the protargol impregnation method [39]. The protargol S™ was manufactured by Polysciences Inc., Warrington, PA (Cat No.: 01070). Drawings were based on free-hand sketches. One subculture of the Hong Kong isolate in this study was deposited in ATCC (American Type Culture Collection; Reg. No.: PRA-270).

### Monitoring individual asymmetric dividers with continuous microscopy

For continuous microscopy of *G. trihymene *reproduction, 50 cultures were established in wheat grain medium (100 × 15 mm plastic Petri dishes each with 3 autoclaved wheat grains in 30 mL autoclaved seawater, 0.2 g/grain, and with ca. 50 tomites in 100 μL stock culture medium as inoculum). The salinity was about 31‰, pH 8.0. All cultures were maintained at room temperature, ca. 23°C. Most asymmetric dividers, which were first observed under a stereomicroscope, were immobile or slowly moving on bottoms of Petri dishes, and their position was marked on the Petri dish bottom. The asymmetric dividers were then observed and followed under an inverted microscope (100-400×; Olympus IX71). To minimize disturbance to asymmetric dividers during continuous multi-day observation, low light intensity and low magnification were used. Asymmetric dividers from 3-7 day-old cultures were continuously isolated with fine pipettes and impregnated with protargol, in order to check the nuclei and infraciliature characters during asymmetric divisions.

### Effect of bacterial concentration on asymmetric division

The Erd-Schreiber soil extract medium added with bacterial suspension has recently been shown to be efficient for culturing *G. trihymene *[40,41] (we believe *Urocryptum tortum *in [40] is a junior synonym of *G. trihymene*, because of their great similarity in living morphology, infraciliature, habitat, as well as the life cycle characteristics). To prepare bacterial suspension, 10 μL stock culture medium without cells was inoculated into 3 mL autoclaved seawater LB medium in test tubes (seawater LB recipe: 12.5 g LB broth in 500 mL autoclaved filtered natural seawater) and cultured at 30°C, 200 rpm, overnight, to maximal growth. The bacteria were harvested by centrifugation at 7378 g in 1.5 mL eppendorf tubes (1 mL bacteria culture in each tube) with a microcentrifuge and the supernatant was removed. Then 1 mL sterile Erd-Schreiber soil extract medium was added to each tube to wash the bacteria pellets, at 7378 g. This washing procedure was repeated twice. Each pellet was finally resuspended with 1 mL soil extract medium and combined in a sterile 50 mL polypropylene conical tube (BD Flacon™).

Bacterial suspensions of 3 mL, 0.3 mL and 0.03 mL were added separately into 3 Petri dishes with sterile soil extract medium to reach a final volume of 30 mL (marked as 1×, 0.1× and 0.01× for each concentration, respectively). It should be noted that the Erd-Schreiber soil extract medium was not a rich medium supporting growth of a large number of bacteria. Four replicates were prepared for each concentration. After each culture was inoculated with about 50 tomites in 100 μL stock culture medium, all 12 cultures were placed on a rocking platform at 3 rpm. Each culture was checked every 12 hours for asymmetric dividers, until 50 hours after the inoculation (preliminary experiments showed that the earliest appearance of asymmetric dividers occurred 50 hours after inoculation with tomites). After 50 hours, all cultures were checked for appearance of asymmetric dividers every two hours until they were first observed in each culture. The first appearance time of asymmetric dividers and tomites was recorded for each culture. Subsequently, all cultures were checked for the presence of asymmetric dividers every 12 hours, until all of them disappeared from each culture. The disappearance time point of asymmetric dividers for each culture was also recorded.

### Amplifying, cloning and sequencing of SSU rDNA

Cells from the stock culture were harvested in one 1.5 mL eppendorf tube with a micro-centrifuge, at 1844 g. Supernatant was removed and the pellet was re-suspended with 20 μL autoclaved seawater. The cell suspension was directly used as DNA template for amplifying the SSU rDNA. Universal eukaryotic primers for SSU rRNA were used: forward 5'-AACCTGGTTGATCCTGCCAGT-3', reverse 5'-TGATCCTTCTGCAGGTTCACCTAC-3' [42]. PCR programs were performed using the iProof™ High-Fidelity PCR kit (Bio-Rad, CA): 1 cycle (98°C, 2 min); 30 cycles (98°C, 10 s; 70°C, 30s; 72°C, 50s); 1 cycle (72°C, 7 min). The PCR products were then purified with the QIAquick gel extraction kit (QIAGEN Sciences, MD) and cloned with the Zero Blunt TOPO kit (Invitrogen, CA). The plasmid DNA was isolated from transformant colonies using the QIAprep spin miniprep kit (Qiagen, CA) and four clones were sequenced with the BigDye terminator kit (Applied Biosystems, CA) on an automated ABI 3130 XL sequencer in the Department of Microbiology and Molecular Genetics, University of Texas Health Sciences Center at Houston.

### Sequence availability and phylogenetic tree reconstruction

The SSU rDNA sequence of *G. trihymene *was deposited in GenBank [GenBank: GQ214552]. The accession numbers of the additional SSU rDNA sequences used in this study were as follows: *Anophryoides haemophila *[GenBank: U51554], *Anoplophrya marylandensis *[GenBank: AY547546], *Cardiostomatella vermiforme *[GenBank: AY881632], *Cohnilembus verminus *[GenBank: Z22878], *Colpoda inflata *[GenBank: M97908], *Cyclidium glaucoma *[GenBank: EU032356], *Entorhipidium pilatum *[GenBank: AY541689], *Gymnodinioides pitelkae *[GenBank: EU503534], *Histiobalantium natans viridis *[GenBank: AB450957], *Hyalophysa chattoni *[GenBank: EU503536], *Metanophrys similes *[GenBank: AY314803], *Miamiensis avidus *[GenBank: AY550080], *Pleuronema coronatum *[GenBank: AY103188], *Pseudocohnilembus hargisi *[GenBank: AY833087], *Schizocalyptra aeschtae *[GenBank: DQ777744], *Schizocaryum dogieli *[GenBank: AF527756], *Uronema marinum *[GenBank: AY551905], *Vampyrophrya pelagica *[GenBank: EU503539].

Sequences were aligned in ClustalW [43] (executed as a plug-in in Geneious Pro 4.0.4 [44]) and adjusted by hand. 1707 nucleotides (positions) were used in the analysis. Maximum likelihood (ML) and parsimony (MP) phylogenetic analyses were performed in PAUP* [45] and Baysian analyses (MB) in Mr. Bayes [46] (both executed in Geneious Pro 4.0.4) using the best fit model as determined by ModelTest [47] (GTR+I+G). Support was determined based on 100 bootstrap replicates (ML and MP) or the posterior probability after one million generations, with an initial 10% burn-in (MB).

### Statistical analysis

Oneway ANOVA analysis (Tukey HSD Test, α = 0.05, JMP 7 software package) was conducted to assess the differences among first appearance time and persistence time of asymmetric dividers in cultures with three different concentrations of bacterial suspension (data was log-transformed into normal distribution).

## Authors' contributions

HL discovered the first asymmetric divider. RAZ and HL designed the study. HL collected the data. RAZ provided reagents and equipment. RAZ and HL analyzed and interpreted the data and wrote the manuscript. Both authors read and approved the final manuscript.
